# Effects of kinship or familiarity? Small thrips larvae experience lower predation risk only in groups of mixed-size siblings

**DOI:** 10.1007/s00265-014-1715-x

**Published:** 2014-04-01

**Authors:** Paulien J. A. de Bruijn, Maurice W. Sabelis, Martijn Egas

**Affiliations:** Institute for Biodiversity and Ecosystem Dynamics, University of Amsterdam, Amsterdam, The Netherlands

**Keywords:** *Frankliniella occidentalis*, *Iphiseius degenerans*, Kin discrimination, Predation risk, Size structure, Non-social insects

## Abstract

In many species of insects, larvae are distributed in an aggregated fashion. As they may differ in size and size matters to predation risk, small larvae may be less likely to fall prey to predators when near large and therefore better-defended larvae. We hypothesize that the small larvae may profit even more when these large larvae are siblings. We tested this hypothesis on kinship-dependent survival in groups of larvae of the Western flower thrips (*Frankliniella occidentalis*) exposed to a predatory mite (*Iphiseius degenerans*). Our experiments showed that small larvae in sibling groups survive significantly better than in non-sibling groups, but only when such groups consisted of a mixture of small and large larvae. To test whether the survival effect we found is due to familiarity of thrips larvae growing up together (i.e. on one leaf), we also measured survival in sibling groups of larvae grown up on different leaves and in non-sibling groups of larvae grown up on the same leaf. These experiments showed an increased survival of small thrips larvae only in groups of sibling larvae from the same leaf. Non-sibling larvae did not show an increased survival when they come from the same leaf. Our results indicated that the increased survival in sibling groups was only partly due to the familiarity effect we tested. Growing up together did not return the same survival effect for non-siblings as it did for siblings. We conclude that growing up together is a necessary but not sufficient condition for discrimination in thrips larvae.

## Introduction

Kin discrimination and its effects on fitness have been well studied in mammals (Silk 2002), birds (Komdeur and Hatchwell 1999), amphibians (Blaustein and Waldman 1992) and social insects (e.g. Queller and Strassmann 1998, but see also Keller 1997). For example, when presented with a predator model (stuffed badger), black-tailed prairie dogs call alarm more frequently when they are in groups with close genetic relatives than when they are in groups without (Hoogland 1983). In this way, kin discrimination serves to direct potentially beneficial behaviour towards relatives, rather than towards unrelated individuals.

In non-social arthropods, kin discrimination received much less attention (Fellowes 1998, for specific examples see: Jasienski et al. 1988; Faraji et al. 2000; Magalhães et al. 2005; Schausberger 2007). Fellowes (1998) identified six behavioural elements in non-social arthropods that could be biased by relatedness: (1) resource exploitation, (2) sex allocation under local mate competition, (3) inbreeding, (4) cannibalism, (5) superparasitism and (6) aggregation when exposed to predation risk, which is the element of interest in this study.

Here, we focus on the effects of aggregation behaviour in response to predation risk. In such a situation, the composition of a group can be important for the survival of individuals, especially when some individuals in a group are more vulnerable to predation than others. Vulnerability often depends on the size of an individual, because many predators attack prey of different sizes. For example, some predators attack only the smallest individuals (Lima and Dill 1990) or individuals from a certain size range (Tonn et al. 1992; Chase 1999). In the former case, smaller prey individuals can experience decreased predation risk in the vicinity of larger individuals because larger prey individuals may actively or inactively hinder the predator before or during an attack on smaller prey individuals. Such decreased in predation risk for smaller individuals may be expected when the relatedness among prey individuals is high enough (conform kin selection, Hamilton 1964, however, see van Veelen 2009). We, therefore, hypothesize that small individuals (i.e. the preferred prey) will experience increased survival when near larger siblings.

To test this hypothesis, we use the Western flower thrips, *Frankliniella occidentalis*. Thrips are suitable for an experimental approach to answer this question, for four reasons. First, the difference in size between first- and second-instar larvae is considerable (factor 1.5 in length and in width, and 1.6 in height, de Bruijn pers. obs.). Second, first- and second-instar thrips larvae occur together in groups on leaves. Third, thrips larvae are preyed upon by many different predators, differing in size, attack rate, and attack success. Fourth, thrips have defensive traits that reduce the attack success of their predators. Upon encounter with a predator, thrips quickly move their abdomen to and fro (here referred to as abdominal swings), trying to hit the predator and, when the threat of predation persists, they release anal droplets that contain an alarm pheromone and cause a predator to retreat and groom (Bakker and Sabelis 1989; de Bruijn et al. 2006). The effectiveness of these traits depends on the size of the predator encountered, as well as on the size of the thrips larvae (Bakker and Sabelis 1987). In this study, we use *Iphiseius degenerans*, a blind predatory mite of c. 0.5 mm that mostly attacks first-instar thrips larvae (c. 0.75 mm; de Bruijn pers. obs.) (Faraji et al. 2001) and has difficulties in attacking second-instar larvae (c. 1.0 mm; de Bruijn pers. obs.). When this predatory mite approaches a thrips larva at its flank, they may wrestle for some time, during which the predator usually tries to lift the thrips from the surface and to feed from the, then defenseless, thrips (de Bruijn pers. obs.). The chance that a first-instar larva survives an attack by a predatory mite is c. 30 %, while this chance is c. 70 % for second-instar larvae (de Bruijn pers. obs). Given these characteristics of the predator–prey system under study, we predict that the first-instar thrips larvae have a higher chance to survive predation by *I. degenerans* when living in groups with second-instar thrips larvae that are siblings.

To test this hypothesis, we measured the survival of thrips larvae under predation in groups with same-sized individuals (all small or all large) and in mixed groups (half small and half large), both for groups where all individuals are siblings and groups where all individuals are non-siblings. To test how thrips discriminate kin, we added treatments with sibling groups where small and big larvae had never encountered each other before, and with non-sibling groups where larvae had grown up together on the same leaf.

## Material and methods

### Thrips

Western flower thrips, *F. occidentalis* (Pergande), were collected from cucumber plants in a commercial greenhouse near Pijnacker, the Netherlands, in February 2006. Thrips were subsequently reared in a climate box (25 °C, 60 % RH, L16:D8) on cucumber leaves, cut to fit in a Petri dish on top of a layer of cotton wool that was put on the bottom of the Petri dish. Once a week, thrips pupae and adults from older leaves of the culture were put on the cucumber leaf, and pollen of *Typha latifolia* was provided on this leaf as additional food for the thrips. From the eggs produced by the adult females, thrips larvae hatched. The emerging pupae and adults were then transferred to a new leaf in a new Petri dish to rear a next generation of thrips. This procedure was repeated to maintain a culture. The laboratory culture usually contained at least 500 individuals, with an occasional dip of c. 200 individuals.

### Predatory mites

The predatory mite, *I. degenerans*, originating from Rabat, Morocco, was reared on a diet of *Typha* pollen in a climate box at 25 °C, 60 % RH and L16:D8. The rearing arenas consisted of a PVC sheet (6 × 15 cm) placed on a wet sponge in a water-containing tray. The edges of the PVC sheet were covered with paper tissue that absorbs water from the sponge underneath. The tissue served as a water source to the predatory mites and as a barrier to prevent escape from the PVC arena. Short threads of cotton placed on the PVC sheet served as a substrate for oviposition by the predatory mites. For the experiments, we used adult females, 8–15 days old since hatching and 0.7 mm in length.

### Experimental setup to measure survival under predation

Adult females were put each on a separate leaf fragment to lay eggs. Four to eight days later, larvae were collected from these leaf fragments. To establish sibling groups, ten larvae were collected from a single leaf fragment (i.e. all offspring from the same mother), whereas to establish non-sibling groups, each of the ten larvae was collected from a different leaf fragment. Because adult female thrips can lay 4–5 eggs per day (van Rijn et al. 1995), our setup enabled us to collect ten similar-sized larvae from one leaf disc as well as from ten different leaf fragments.

For the survival experiments, arenas were prepared in the following way. A leaf disc (∅ 24 mm), excised from the cotyledon of a cucumber plant, was put on a layer of wet cotton wool in a plastic cup (hight 70 mm, ∅ 66 mm). The cup had a lid with an opening covered with gauze, to prevent the arena from becoming too humid. Ten thrips larvae (either ten first-instar larvae, or ten second-instar larvae, or five first-instar larvae or five second-instar larvae), either all sibling or all non-sibling as described above, were put on the leaf disc and a single *I. degenerans* predator was added. From September 2006 until May 2007, for 5 days, twice per day (morning between 10.00 and 11.00, and afternoon between 6.00 and 7.00), the thrips larvae that were present and alive were counted. Because we were unable to observe the larvae in our experiment continuously, we do not know whether larvae that were missing were killed by the predator or drowned in the water barrier surrounding the leaf disc but we considered them to be consumed by the predator. The instar of the larvae (first or second) was also noted. In the groups of thrips larvae with mixed sizes, first-instar larvae that developed into the next instar were scored as second-instar larvae. When a thrips reached the pre-pupal state, it was removed and censored as a survivor. This was done because under natural conditions pre-pupal thrips leave the plant. Any replicate where a predator had died before the end of the experiment was discarded. In total, at least 20 replicates of each treatment were scored. As a control for causes of death other than predation, we performed the same experiments without a predatory mite. All survival experiments were performed in a climate room (25 °C and 60 % humidity at L16:D8).

### Experimental setup to test for kin discrimination

To test the possible mechanisms thrips larvae use to discriminate kin, we conducted a test similar to the one used for survival under predation, with a few modifications. These experiments were conducted in the laboratory (±21 °C and ±50 % humidity, natural daylight), in the period from March to May 2013. Five first-instar thrips larvae and five second-instar thrips larvae were introduced on an arena as described above with an *I. degenerans* predator, and then the surviving first-instar and second-instar larvae were counted 6 h later. Compared to the ‘survival under predation’ setup, we composed two more groups: ‘sibling-different-leaf’ (SDL) and ‘non-sibling-same-leaf’ (NSSL). For this, we put single adult females on a separate leaf discs and moved the females after 1, 2 and 3 days to new, clean, leaf discs. In this way, we established three groups of larvae, all siblings, which had never encountered a sibling of the other groups before. We created an SDL group by selecting five first-instar larvae from the youngest group of siblings and a total of five second-instar larvae from the two older groups of siblings. As before, we selected similar-sized larvae, but due to the different age of second-instar siblings on different leaf fragments, their variation in size was slightly higher than in the SSL treatment. An NSSL group was created by putting 10–15 females on a leaf fragment. To treat the NSSL group similar as the SDL group, we picked NSSL females up and put them back on the same leaf fragment on the same days as adult females for the SDL treatment were moved to different leaf fragments. After 8 days, we randomly took five similar-sized first-instar larvae and five similar-sized second-instar larvae from these leaf fragments. It was not possible for us to check the relatedness of the ten larvae, but we can reasonably assume most larvae in each replicate were non-siblings. We composed the groups with sibling larvae from one leaf fragment and non-sibling larvae from different leaf fragments as described above (henceforth called ‘sibling-same-leaf’ (SSL) and ‘non-sibling-different-leaf’ (NSDL)), with one addition: thrips females were picked up and put back on the leaf fragment on the same days as adult females for the SDL treatment were moved to different leaf fragments. Because the treatments SSL and NSSL differed in kinship of the thrips larvae, as well as the density of the thrips on the leaf fragment, and SDL and NSDL differed in kinship of the thrips larvae as well as the number of leaf fragments they were collected from, we compare only SSL with SDL and NSSL with NSDL to test for the effect of growing up on the same leaf.

### Statistics

To compare the survival among treatments of each of the two experiments, we applied a GLM assuming a Poisson distribution of the number of dead larvae. Effects of treatment were tested in this GLM using a one-way ANOVA. All analyses were done using the open source program R (R Development Core Team 2010).

## Results

### Kin survival

**Fig. 1:**
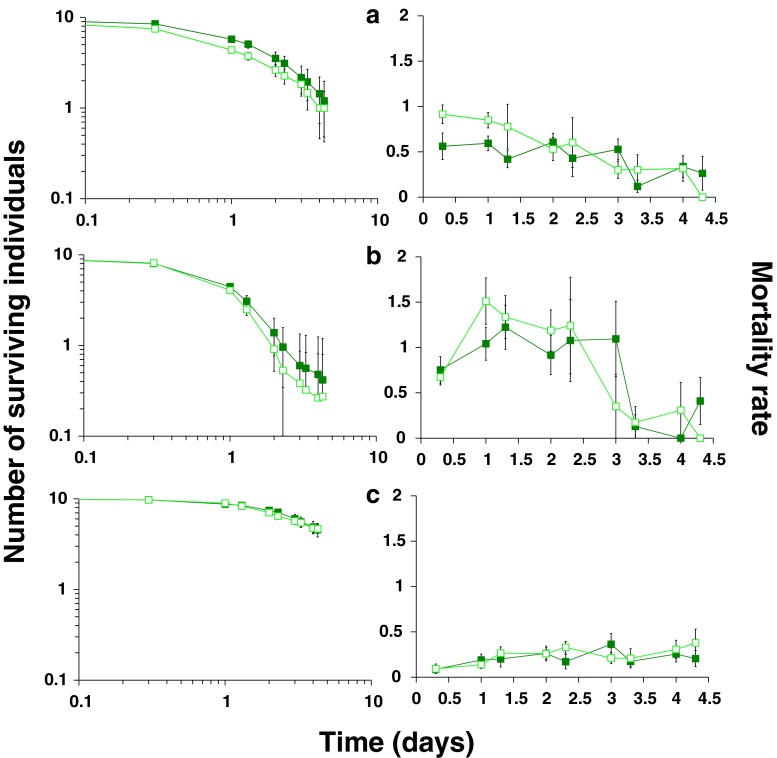
Survival (*left panels*) and mortality rate (*right panels*) of thrips larvae in the presence of the predatory mite *I. degenerans*. On the *x*-axes is the time in days. On the *y*-axis of the *left panels* is the average number of surviving thrips larvae during 4.3 days in sibling groups (*dark green filled boxes*) or non-sibling groups (*light green open boxes*) that were composed of **a** five first-instar and five second-instar larvae (*N* = 20 for sibling groups, 19 for non-sibling groups), **b** ten first-instar larvae (*N* = 31 for sibling groups, 35 for non-sibling groups) and **c** ten second-instar larvae (*N* = 19 for sibling groups, 19 for non-sibling groups). To facilitate comparison of the survival data, the *right panels* show the corresponding average mortality rates (day^−1^) calculated from the survival measurements. Note the difference in mortality rate at the start of the experiment in panel **a**. *Error bars* show standard errors

When first- and second-instar larvae were put together on a leaf disc with a predatory mite, the difference in survival of larvae between sibling and non-sibling groups was significant after 8 h (Fig. 1a, left panel; GLM deviance = 4.2, df = 1, *p* < 0.05). The difference in survival between siblings and non-siblings in the groups starting with ten first-instar larvae or ten second-instar larvae was not significant (Fig. 1b, c, left panels; GLM, first-instar larvae; deviance = 0.06, df = 1, *p* = 0.81 and second-instar larvae; deviance = 0.003, df = 1, *p* = 0.95). In the treatment with first- and second-instar larvae and the treatment with exclusively first-instar larvae, most thrips did not survive up to the end of the experiment, but in the treatment with exclusively second-instar larvae, on average 4.6 of the ten thrips larvae survived.

**Fig. 2:**
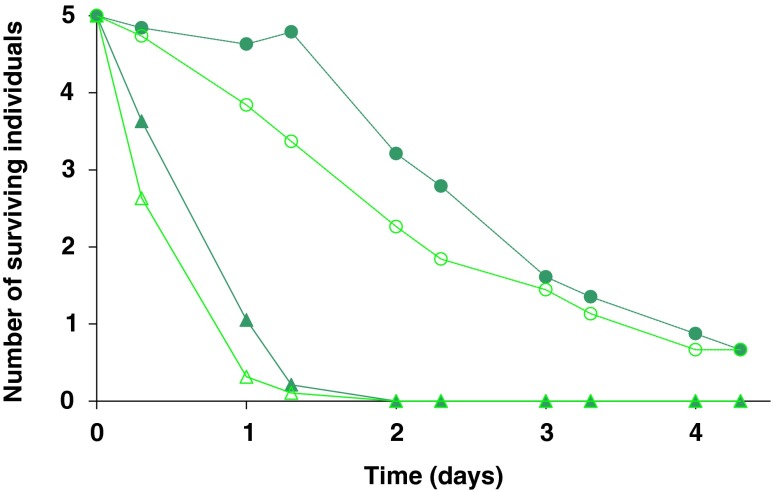
Survival of thrips larvae in mixed-size groups of siblings or non-siblings during 4.3 days. The data are the same as in Fig. 1a, but displayed separately for first-instar larvae and second-instar larvae. On the *x*-axis is the time in days, on the *y*-axis the fraction of surviving individuals. First-instar larvae are presented with *triangles* and a *solid line*, second-instar larvae with *circles* and *dotted line*; sibling groups in *dark green* and non-sibling groups in *light green*

To further analyse the difference in survival of thrips larvae within mixed groups (as in Fig. 1a, left panel), survival of first- and second-instar larvae is shown separately in Fig. 2. The largest difference in survival between siblings and non-siblings was found in the first-instar larvae, and this difference became manifest after c. 8 h. Here, the difference in survival of first-instar larvae was significant (Fig. 2; GLM, deviance = 4.3, df = 1, *p* < 0.05), but the difference in survival of second-instar larvae was not significant (Fig. 2; GLM, deviance = 0.1, df = 1, *p* = 0.8).

After 1 or 2 days, all first-instar larvae had developed into (and were therefore counted as) second-instar larvae. This explains the increase in the number of second-instar sibling larvae after 1.3 days. Figure 2 shows that the difference in survival found for this mixed-size group is mostly explained by the difference in survival of the first-instar larvae.

**Fig. 3:**
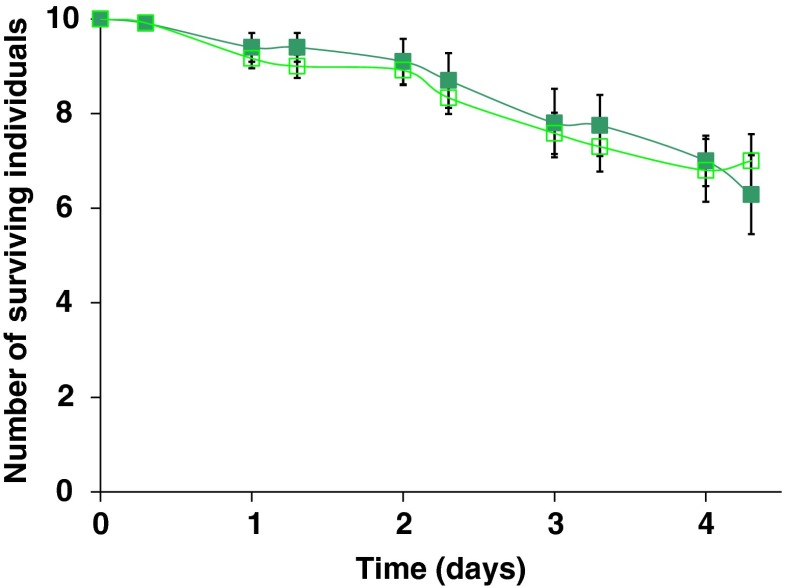
Survival of thrips larvae in mixed-size groups of siblings or non-siblings during 4.3 days in absence of predation. On the *x*-axis is the time in days, on the *y*-axis the number of surviving individuals. *Error bars* show standard errors. *N* = 12 for both sibling groups (*dark green filled boxes*) and non-sibling groups (*light green open boxes*). *Error bars* show standard errors

As a control for causes of death other than the presence of predator in the mixed-size groups, we repeated the experiment, but now without a predator (Fig. 3). In this control experiment, there was no significant difference between sibling and non-sibling individuals after 8 h (GLM; deviance = 0.003, df = 1, *p* = 0.95). We found that after 4.3 days, 6–7 individuals survived instead of less than two in the experiments with a predator.

### Kin discrimination

**Fig. 4:**
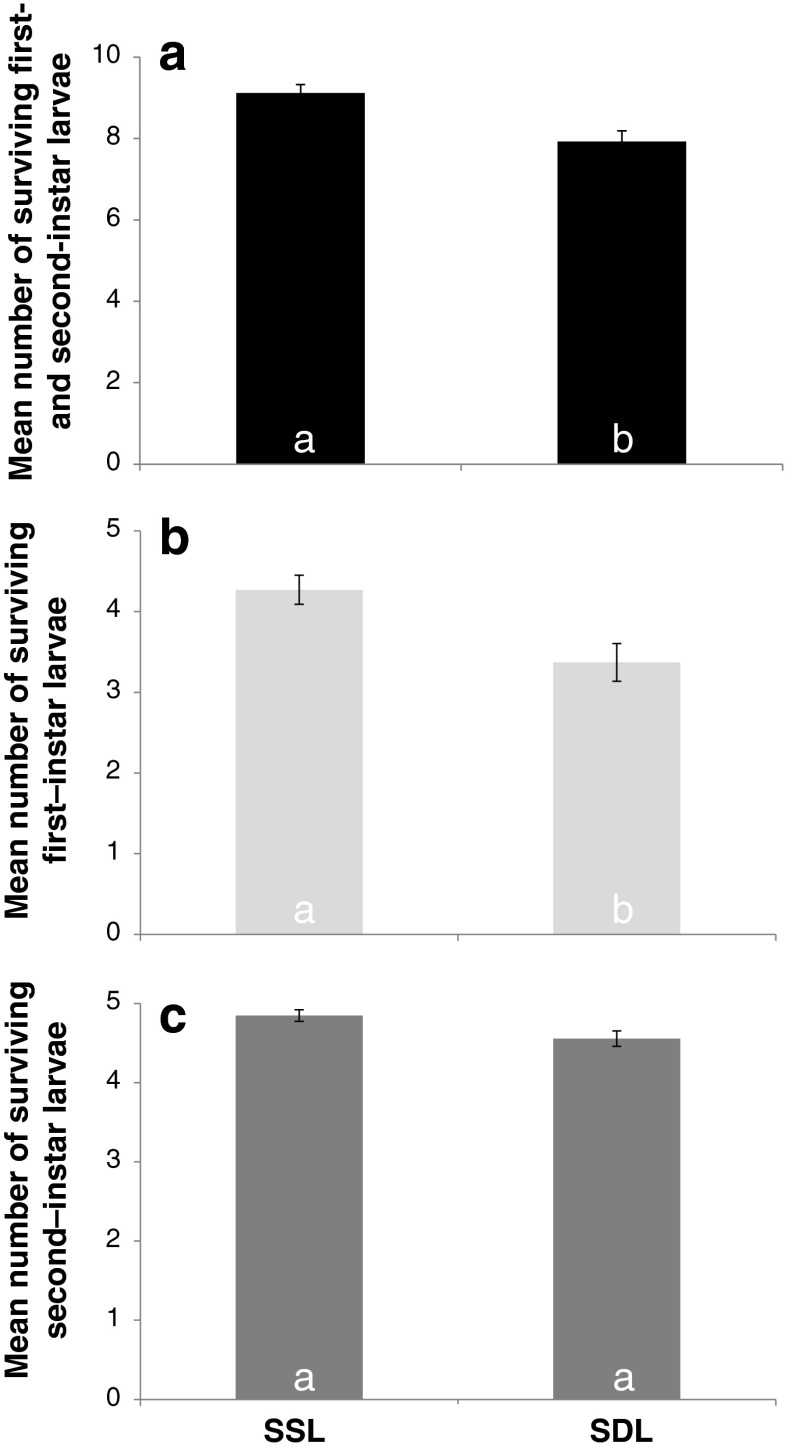
Survival of sibling thrips larvae in mixed-size groups after 6 h of exposure to predation. On the *x*-axis are the treatments, *SSL* refers to sibling same leaf, *SDL* refers to sibling different leaf. On the *y*-axis is the number of surviving individuals. The number of replicates is 26 for SSL, 27 for SDL. *Error bars* show standard errors. **a** Survival of the first- and second-instar larvae together. **b** Survival of the first-instar larvae and **c** survival of the second-instar larvae

**Fig. 5:**
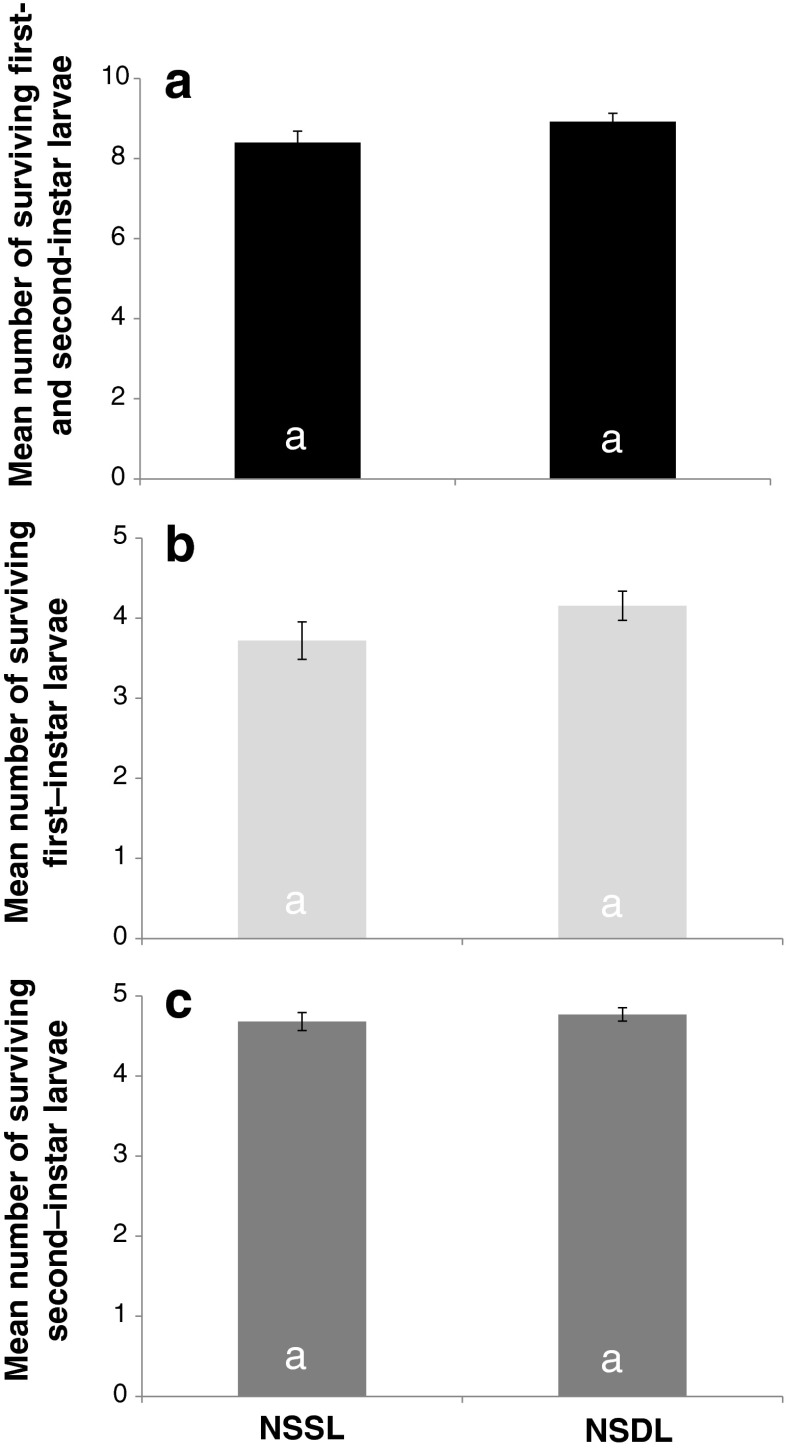
Survival of non-sibling thrips larvae in mixed-size groups after 6 h of exposure to predation. On the *x*-axis are the treatments, *NSSL* refers to non-sibling same leaf, *NSDL* refers to non-sibling different leaf. On the *y*-axis is the number of surviving individuals. The number of replicates is 25 for NSSL, 26 for NSDL. *Error bars* show standard errors. **a** Survival of the first- and second-instar larvae together. **b** Survival of the first-instar larvae and **c** survival of the second-instar larvae

For sibling thrips larvae, we found a higher survival when the larvae have encountered each other before (Fig. 4; GLM, first- and second-instar larvae together, deviance = 13.0, df = 1, *p* < 0.01). For non-sibling larvae, we did not find this difference (Fig. 5; GLM, first- and second-instar larvae together, deviance = 2.6, df = 1, *p* = 0.1).

## Discussion

When thrips larvae of different sizes occur in groups, small sibling larvae survive better than small non-sibling larvae. However, kinship does not influence larval survival in uniform-size groups. What causes the increased survival in mixed-size sibling groups? First-instar larvae are consumed more frequently by *I. degenerans* than second-instar larvae (Fig. 1b, c). The difference in survival in the mixed-size groups is mainly due to increased survival of first-instar larvae in the sibling group (Fig. 2). This difference becomes manifest after half a day. Thereafter, also a difference in second-instar larvae emerges, but this is because from day 2 on first-instar larvae develop into second-instar larvae, and subsequently scored as second-instar larvae. Because more first-instar larvae survive, we find more second-instar larvae from day 2 onwards. In the absence of a predator, survival is high for both sibling and non-sibling groups (Fig. 3). Hence, the data support our hypothesis that the presence of second-instar larvae increases the survival of sibling first-instar larvae under predation by *I. degenerans*. We are not aware of other studies testing if vulnerable prey experience increased survival when in the vicinity of less vulnerable siblings. This kind of kinship effects, however, may occur generally in prey species with stages that vary in vulnerability to predators.

In these experiments, we find a very clear effect of kinship, despite the fact that the adult thrips females that are used to create groups of sibling thrips, but also non-sibling thrips, come from the same culture that we had maintained for multiple generations in our lab. This means that non-sibling thrips in our experiment are probably more related than non-sibling thrips in the field. Together, this leads to two mutually non-exclusive predictions: (1) the effect of kinship would be even more pronounced with individuals from a natural population, or (2) thrips larvae recognise siblings when they have grown up with them, i.e. a familiarity effect.

We provide evidence that familiarity does contribute to the effect of kinship on larval survival. Sibling thrips larvae survive being near a predatory mite better when they all come from one leaf fragment than when the larvae have grown up on one of the three different leaf fragments (Fig. 4). This suggests that thrips larvae need to learn about the other sibling thrips before they can discriminate them as kin. For non-sibling larvae, we find no difference in survival between groups that come from one leaf fragment and groups that come from ten leaf fragments (Fig. 5) even though the groups of non-sibling larvae that come from one leaf fragment might contain some sibling individuals. This suggests that growing up on the same leaf fragment is not enough for thrips larvae to discriminate kin from non-kin.

Our results show that there is some form of kin discrimination in thrips larvae. This adds to the body of literature on kin recognition in non-social arthropods (Fellowes 1998; Gherardi et al. 2012). The way thrips recognise each other determines whether thrips help genuine kin or not. Individuals can recognise kin by cues that are determined by the genotype of this sibling or by cues that come from the shared environment of the two siblings. There are many mechanisms that would allow recognition, both from genetic and environmental cues (for example cuticular hydrocarbons (CHCs), see Singer 1998; for a specific example, see Weddle et al. 2013a). In ants, examples of cues from the environment that influence nest mate recognition include diet, ambient odours, and nest material (Obin 1986). If recognition occurs through environmentally determined cues, the cue is indirect and hence individuals could fail to recognise siblings from a different location or individuals could fail to distinguish between kin and non-kin from a common location. However, if recognition occurs through genotypically determined cues, individuals recognise kin by a direct cue, independent of a common environment (as shown for CHCs in Lihoreau et al. 2007). Gerlach et al. (2008) showed that for zebrafish, both genotypically and environmentally determined cues are necessary for kin recognition. Larvae are sensitive to olfactory cues of kin individuals in a specific time frame during development, in which imprinting on kin odours may occur. However, when larvae are exposed to non-kin cues in this time frame, imprinting did not occur. Our findings for thrips larvae are similar. Survival of sibling thrips larvae is higher when they have grown up together, but this effect is not found in non-sibling thrips larvae. These results suggest that kin recognition in thrips larvae requires environmentally as well as genotypically determined cues. In particular, it may be that the enhanced effect of growing up together is mediated by self-referent phenotype matching, i.e. an individual may only imprint on kin odours when these are sufficiently similar to those of itself. Self-referencing is a widespread mechanism in arthropods, often involving CHCs as cues (Weddle et al. 2013b), but it has not yet been studied in thrips. Several studies have characterized CHCs for the Western flower thrips (Gołębiowski et al. 2007; Zhao et al. 2011), and one of these CHCs is known to act as a male recognition pheromone (Olaniran et al. 2013). We, therefore, hypothesize that Western flower thrips are capable of kin recognition by self-referent phenotype matching using CHCs.
